# Effects of mutations and deletions in the human optineurin gene

**DOI:** 10.1186/2193-1801-3-99

**Published:** 2014-02-19

**Authors:** Sanja Turturro, Xiang Shen, Rajalekshmy Shyam, Beatrice YJT Yue, Hongyu Ying

**Affiliations:** Department of Ophthalmology and Visual Sciences, University of Illinois at Chicago, College of Medicine, 1855 W Taylor Street, Chicago, IL 60612 USA

**Keywords:** Optineurin, Glaucoma, Amyotrophic lateral sclerosis, Mutations, Deletion fragments, Phenotypes

## Abstract

Optineurin is a gene associated with normal tension glaucoma (NTG) and amyotrophic lateral sclerosis (ALS). Foci formation and functional consequences including Golgi fragmentation, impairment of vesicle trafficking and apoptosis were observed previously upon overexpression and/or mutation of optineurin.

In the current study, a total of 15 GFP tagged constructs that included NTG (E50K and 2 bp-AG insertion), ALS (exon 5 deletion, R96L, Q398X, and E478G) and non-disease (L157A and D474N) associated mutants and a series of deletion fragments were cloned into mammalian expression vectors and transfected into RGC5 and/or Neuro2A cells to evaluate whether their expression confer the optineurin phenotypes. The cells were monitored for foci formation and stained by immunofluorescence with anti-GM130 to analyze the Golgi integrity. Transferrin uptake experiments were performed to evaluate the protein trafficking process and apoptosis was assessed with the active caspase 3/7 detection kit. We demonstrated that cells expressing E50K and R96L optineurin exhibited all of the optineurin phenotypes. Q398X mutant did not induce foci formation, but triggered Golgi fragmentation, impairment of transferrin uptake and increase in apoptosis. The 2 bp-AG insertion mutant had a nuclear localization, compromised the transferrin uptake and strongly induced apoptosis. The foci formation, which might not predict the rest of the phenotypes, appeared to require both the leucine zipper and ubiquitin binding domains of the optineurin sequence. Interactions of optineurin with proteins including Rab8, myosin VI, huntingtin and transferrin receptor might directly determine whether the Golgi and protein trafficking phenotypes would be manifested. Examination of mutants and deletion fragments located at various sites of optineurin gene provide clues as to what regions of the gene may play a critical role in the development of pathologic consequences.

## Introduction

Optineurin, a 67-kDa protein, has attracted much attention in the neuroscience fields in recent years. It was first isolated in 1998 by Li et al. (1998) in yeast 2-hybrid screen and has been shown subsequently (Schwamborn et al. 2000) to have a strong homology to NF-κB essential molecule (NEMO). In 2002 (Rezaie et al. 2002), the optineurin or “optic neuropathy inducing” gene was identified to be a candidate gene of primary open-angle glaucoma (POAG), the most common form of glaucoma, one of the leading causes of irreversible bilateral blindness worldwide. POAG, characterized by degeneration of retinal ganglion cells and progressive axonal and visual field loss, is age-related and frequently associated with increased intraocular pressure (Stamer and Acott 2012). It is genetically heterogeneous, caused by several susceptibility genes (Allingham et al. 2009; Fingert 2011; Wiggs 2012) and also environmental factors (Wiggs 2012). Optineurin was found to be linked in particular to normal tension glaucoma (NTG) (Rezaie et al. 2002; Sarfarazi and Rezaie 2003), a subtype of POAG.

More recently, mutations in optineurin were also reported to be associated with amyotrophic lateral sclerosis (ALS) (Maruyama et al. 2010; Deng et al. 2011). Optineurin was noted to be localized in pathological structures in ALS, neurofibrillary tangles and dystrophic neuritis in Alzheimer’s disease, as well as Lewy bodies and Lewy neuritis in Parkinson’s disease (Osawa et al. 2011). In addition, optineurin was identified as one of the genetic risk factor for Paget’s disease of bone (Albagha et al. 2010; Chung et al. 2010).

**Figure 1:**
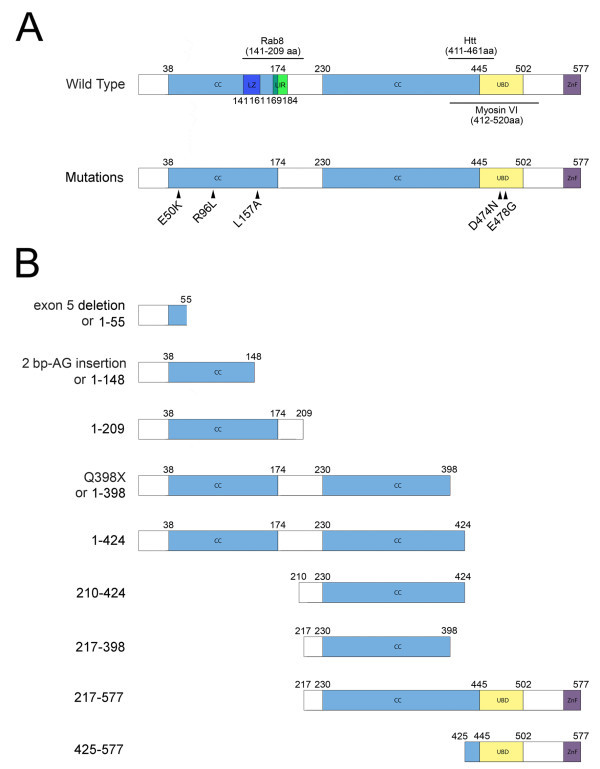
**Schematic representation of mutants and deletion fragments examined in the current study. A**. The full length of wild type (1–577) optineurin protein consists of a leucine zipper (LZ, dark blue) domain, LC3 interacting (LIR, green) motif, coiled-coil (CC, blue) domains, an ubiquitin binding domain (UBD, yellow) and a zinc finger (ZnF, purple) motif. Optineurin mutations are indicated. E50K is associated with NTG. R96L and E478G are associated with ALS. L157A and D474N mutations have not been linked to any diseases so far. **B**. Deletion fragments of optineurin. The glaucoma-associated 2 bp-AG insertion mutation, ALS-associated exon 5 deletion mutation and Q398X mutant result in truncation of the protein into a 1–148, 1–55 fragment and 1–398 fragment respectively and are therefore categorized as deletion fragments.

The human optineurin gene codes for a 577-amino acid protein (Li et al. 1998). The protein consists of a NEMO-like domain, leucine zipper motif, multiple coiled-coil motifs, an ubiquitin binding domain (UBD), a microtubule associated protein 1 light chain 3 (LC3)-interacting motif, and a carboxyl (C)-terminal zinc finger (Figure 1) (Ying and Yue 2012). Optineurin is a cytosolic protein that is not secreted (Ying et al. 2010). It is expressed in many non-ocular tissues such as the brain and the heart as well as in ocular tissues including the retina and the trabecular meshwork (Schwamborn et al. 2000; Sarfarazi and Rezaie 2003; Ying and Yue 2012).

Optineurin has been shown to be a negative regulator of the NF-κB pathway (Schwamborn et al. 2000; Zhu et al. 2007; Sudhakar et al. 2009; Nagabhushana et al. 2011; Akizuki et al. 2013) and a player in mitotic progression (Kachaner et al. 2012a, b). It has also emerged as an autophagy receptor (Wild et al. 2011; Tumbarello et al. 2012,2013). Optineurin is found necessary for optimal activation of TANK binding kinase 1 and interferon regulatory factor 3 in immune cells (Sakaguchi et al. 2011; Munitic et al. 2013) and is noted in addition to be a player in antiviral immune response (Mankouri et al. 2010). This protein is moreover demonstrated to interact and/or form complex with proteins including Rab8, huntingtin (Htt), myosin VI, and transferrin receptor (TfR). The binding sites with Rab8, Htt, and myosin VI reside respectively, between 141–209, 411–461, and 412–520 amino acid residues of optineurin. All these interacting partners are known to have a role in membrane trafficking pathways (Hattula and Peranen 2000; Sahlender et al. 2005; Au et al. 2007; del Toro et al. 2009) and such interactions may be the basis why optineurin is involved in regulation of protein trafficking (Hattula and Peranen 2000; Sahlender et al. 2005; del Toro et al. 2009; Nagabhushana et al. 2010; Park et al. 2010).

In patients with NTG, mutations including Glu50Lys (E50K) and 691_692ins AG (2 bp-AG insertion) have been identified (Rezaie et al. 2002). E50K is a missense mutation and 2 bp-AG insertion is a nonsense mutation that leads to truncation of optineurin protein by 76% (Rezaie et al. 2002). Three mutations in the gene encoding optineurin in Japanese familiar or sporadic ALS patients have also been reported (Maruyama et al. 2010) which include a homozygous deletion of exon 5, a homozygous nonsense Gln398 stop (Q398X) and a heterozygous missense Glu478Gly (E478G). Additional mutations associated with ALS have been identified, such as Arg96Leu (R96L) mutation in French families of ALS patients (Millecamps et al. 2011).

It has been previously reported that overexpression of wild type optineurin resulted in formation of bright granular or punctate structures, termed foci, and fragmentation of the Golgi (Park et al. 2006; Ying et al. 2010). In addition, impairment of transferrin uptake (Park et al. 2010) and apoptosis (Koga et al. 2010) were observed. In cells expressing E50K, all these phenotypes were manifested to a greater degree than in the wild type (Park et al. 2006,2010; Nagabhushana et al. 2010; Ying et al. 2010). By contrast, cells expressing Leu157Ala (L157A) and Asp474Asn (D474N) optineurin mutations, evaluated by Park et al. (2010) and Nagabhushana et al. (2010) respectively, showed minimal foci formation and unaltered transferrin uptake. Neither mutation, to date, has been linked to any diseases.

The present study was undertaken to determine whether the expression of a number of optineurin mutations and deletion fragments in cell lines RGC5 and/or Neuro2A would result in the above-mentioned optineurin phenotypes that include foci formation, Golgi fragmentation, compromised transferrin uptake and increased apoptotic activity. A total of 15 green fluorescence protein (GFP) tagged constructs that included wild type optineurin, and NTG (E50K and 2 bp-AG insertion), ALS (exon 5 deletion, R96L, Q398X, and E478G) and non-disease (L157A and D474N) associated mutants as well as various deletion fragments were prepared. These constructs were transfected into cells and the ensuing biological consequences were evaluated.

Missense mutations E50K and R96L are located in the N-terminal coiled-coil domain of optineurin while D474N and E478G are in the C-terminal UBD domain. By computer analysis (http://2zip.molgen.mpg.de/), the change of Leu^157^ to Ala in the optineurin sequence may lead to obliteration of the leucine zipper. The nonsense 2 bp-AG insertion presumably induces a premature stop codon that leads to truncations of the optineurin protein by 76%, yielding a 1-148 fragment. With exon 5 deletion, the resulting transcript would also be expected to translate into a truncated protein (1–55) with only 55 amino acids in length (Ying and Yue 2012). Q398stop (Q398X) mutation in addition would cause truncation, yielding a fragment with 398 amino acid residues (1–398). These 3 mutations or deletion fragments with truncated C-terminal UBD domains along with fragments 1–209, 1–424, 210–424, 217–398, 217–577, and 425–577 lack either the N-terminal coiled-coil, the leucine zipper, the C-terminal coiled-coil, and/or the UBD sequences. A schematic representation of the mutations and fragments is shown in Figure 1.

## Materials and methods

### Cell cultures

RGC5 cells were obtained from the departmental core facility at the University of Illinois at Chicago, deposited by Dr. Paul Knepper (Choi et al. 2005) and generously provided originally by Dr. Neeraj Agarwal (Aoun et al. 2003; Agarwal 2013). RGC5 cells were grown in Dulbecco’s modified Eagle’s medium (DMEM) supplemented with 10% fetal bovine serum (FBS) and antibiotics (Park et al. 2007; Koga et al. 2010; Ying et al. 2010; Shen et al. 2011). Mouse neuronal Neuro2A cells were obtained from Dr. Chunjiang Yu at the University of Illinois at Chicago (Chakrabarti et al. 2013). Neuro2A cells were cultured under conditions similar to RGC5 cells in DMEM supplemented with 10% FBS.

### Plasmid constructs

Expression vectors with enhanced GFP (EGFP) tagged at the C-terminus of wild type, E50K, and L157A optineurin, pOPTN_WT_-EGFP, pOPTN_E50K_-EGFP and pOPTN_L157A_-EGFP, respectively, were constructed as previously described (Park et al. 2006; Ying et al. 2010; Ying and Yue 2012). Mutant constructs pOPTN_R96L_-EGFP, pOPTN_D474N_-EGFP, and pOPTN_E478G_-EGFP were additionally made based on pOPTN_WT_-EGFP by site-directed mutagenesis employing the QuikChange II Site-Directed Mutagenesis kit from Stratagene (La Jolla, CA) or the GeneTailor™ Site-Directed Mutagenesis System from Invitrogen (Grand Island, NY). Primers used were listed in Table 1 with point mutations in boldface. Expression vectors of fragments of optineurin including pEGFP-OPTN_WT_, pEGFP-OPTN_exon 5 del_, -OPTN_1-209_, -OPTN_1-424_, -OPTN_210-424_, and -OPTN_425-577_ were made by polymerase chain reaction (PCR) amplification using primers listed in Table 1 and subsequent subcloning into pEGFP-C1 vector (Clontech, Mountain View, CA). They were tagged with EGFP at the amino (N)-terminus. pEGFP-OPTN_2 bp-AG insertion_ and pEGFP-OPTN_Q398X_ were made by site-directed mutagenesis based on pEGFP-OPTN_WT._ Constructs pEGFP-OPTN_217-398_ and pEGFP-OPTN_217-577_ were made by digestion of plasmids pEGFP-OPTN_Q398X_ and pEGFP-OPTN_WT_ with *BglII*, gel purification and self-ligation. All the constructs were verified by sequence analyses.

**Table 1:** **Primer sequences**

Expression vector	Sense primer	Antisense primer
pOPTN_R96L_-EGFP	AAAGAAGCAAAAGAGC**T**TCTAATGGCCTTG	GCTCTTTTGCTTCTTTGCTCTGTATCTC
pOPTN_D474N_-EGFP	CAGATGGAAGTTTACTGTTCT**A**ATTTTCATGCTGAAA GAGCAGC	GCTGCTCTTTCAGCATGAAAATTAGAACAGTAAACTT CCATCTG
pOPTN_E478G_-EGFP	CTGATTTTCATGCTG**G**AAGAGCAGCGAGAG	CAGCATGAAAATCAGAACAGTAAACTTC
pEGFP-OPTN_2 bp-AG_	CTGAGGACCCCACTGAT**AG**GACTCCAGGCT	ATCAGTGGGGTCCTCAGATGACCTTTCT
pEGFP-OPTN_Q398X_	GACACACAACAAGCTTCTT**T**AAGAACATAATA	AAGAAGCTTGTTGTGTGTCATCTGTAGC
pEGFP-OPTN_wt_	AAA *GAA TTC* AAT GTC CCA TCA ACC TCT CAG	TTT *TCT AGA* TTA AAT GAT GCA ATC CAT CAC
pEGFP-OPTN_1-55_	AAA *GAA TTC* AAT GTC CCA TCA ACC TCT CAG	TTT *TCT AGA* TCA GTG GGG TCC TTT CAG CTG GTG GTT CTC
pEGFP-OPTN_1-209_	AAA *GAA TTC* AAT GTC CCA TCA ACC TCT CAG	TTT *TCT AGA* TTA CGT GCC AGT GGA GAC TGT TC
pEGFP-OPTN_1-424_	AAA *GAA TTC* AAT GTC CCA TCA ACC TCT CAG	TTT *TCT AGA* TTA CTT CAG CAC TGC CCT GTC CA
pEGFP-OPTN_210-424_	AAA *GAA TTC* AAT GGC ATT GTC TAA ATA TAG GAG	TTT *TCT AGA* TTA CTT CAG CAC TGC CCT GTC CA
pEGFP-OPTN_210-577_	AAA *GAA TTC* AAT GGC ATT GTC TAA ATA TAG GAG	TTT *TCT AGA* TTA AAT GAT GCA ATC CAT CAC
pEGFP-OPTN_425-577_	AAA *GAA TTC* AAT GGA ACT GAG TGA AAA ACT GGA	TTT *TCT AGA* TTA AAT GAT GCA ATC CAT CAC

Primer sequences used for constructing GFP-tagged optineurin mutant and deletion fragment expression plasmids are listed. The mutated nucleotides are labeled in boldface. The restriction enzyme sites that were used in the cloning are in italics.

### Immunofluorscence staining

RGC5 or Neuro2A cells plated (7000 cells/well) on Lab-Tek 8-well CC2 glass chamber slides (NalgeNunc, Rochester, NY) were transfected with Lipofectamine LTX transfection reagent (Invitrogen) for 18 hours and fixed with 4% paraformaldehyde for 15 min. After permeabilization in 0.2% Triton X-100 for 4 min, the cells were blocked for 1 hour with 3% bovine serum albumin (BSA) and incubated at room temperature for 1 hour with mouse monoclonal anti-GM130 primary antibody (1:200, BD Biosciences, San Jose, CA). After a further 1 hour incubation with Cy3-goat anti-mouse IgG (1:200; Jackson ImmunoResearch Laboratories, West Grove, PA), the slides were mounted in Vectashield mounting solution (Vector Laboratories, Burlingame, CA) containing 4′,6-diamidino-2-phenylindole (DAPI). The fluorescence was visualized on an Axioscope (Carl Zeiss MicroImaging, Thornwood, NY) with a 63× oil objective. Approximately 20 images were acquired for each specimen.

The images were evaluated for formation of foci which are bright, granular or punctate structures located in the perinuclear region of the cell (Park et al. 2006; Ying et al. 2010). Additionally, the percentage of cells with fragmented Golgi was determined. Golgi fragmentation was defined as the appearance of disconnected, small and round Golgi fragments dispersed in the cells (Park et al. 2006). Cells with compromised Golgi were counted and the percentage of cells containing fragmented Golgi relative to the total number of transfected cells was calculated. A minimum of 40 cells were evaluated for each expression vector per experiment with the exception of D474N and 2 bp-AG insertion constructs with which only approximately 20 cells were examined (due to the low number of transfected cells). Four independent experiments were performed. Results were presented as the average of the 4 experiments (mean ± SEM).

### Transferrin uptake

RGC5 cells plated on Lab-Tek 8-well CC2 glass chamber slides were transfected for 18 hours. Thereafter, the cells were washed with phosphate buffered saline (PBS) and incubated for 1 hour in serum-free DMEM with 0.2% BSA (DMEM-BSA) to deplete serum. The cells were then incubated with DMEM-BSA containing 25 μg/ml of Texas red-transferrin (TR-Tf) (Invitrogen) at 37°C for 0 or 15 min, placed on ice, and washed 3 times with cold PBS containing 0.2% BSA, 1 mM CaCl_2_ and 1 mM MgCl_2_. Following a wash in cold acid buffer containing 0.2 M acetic acid and 0.5 M NaCl and a rinse with ice-cold PBS, the cells were fixed in 4% paraformaldehyde and mounted. Images were acquired with Leica SP2 confocal system (Leica Microsystems, Bannockburn, IL) with a 40× dry objective using sequential scanning to minimize the bleed through.

The uptake of TR-Tf was quantified as described by Park et al. (2010). In brief, the outline of single cell was drawn and using Leica confocal software, the average fluorescence intensity of TR-Tf inside the cell was measured. A region without any fluorescence, which served as the background, was subtracted from the red fluorescence intensity in the cell. The resulting intensity was multiplied by the area of the cell for the total intensity. The values for at least 40 transfected and 40 non-transfected cells in approximately 10 different visual fields were averaged. Five sets of independent experiments were performed. The intensity of TR-Tf for each construct was presented as the average (mean ± SEM) of 5 experiments.

### Apoptosis

Apoptosis was evaluated using the Biomol CV-caspase 3 and 7 detection kit (Enzo Life Sciences, Inc, Farmingdale, NY). The kit utilizes a fluorophore, cresyl violet, coupled to the C-terminus of the optimal tetrapeptide recognition sequences for caspase 3 and 7, DEVD (CR(DEVD)_2_) (Koga et al. 2010). Once target sequences are cleaved by the activated enzymes, red fluorescence throughout the cell is visualized, indicative of apoptotic activity. Briefly, cells on chamber slides transiently transfected for 48 hours were incubated with [CR(DEVD)_2_] in DMEM for 60 min. Following incubation with Hoechst stain for 5 min, the cells were fixed in 4% paraformaldehyde. The slides were mounted and imaged immediately with fluorescence microscopy using a 40× oil objective. Images from 20 randomly selected areas were acquired.

Approximately 60 cells for each construct per experiment were evaluated for apoptotic activity. The number of transfected cells with positive caspase 3/7 activity (red and green fluorescence) and total number of transfected cells (only green) were counted. The percentage of apoptotic cells was calculated from these numbers. Five independent experiments were performed. Data from 2 complete experiments were averaged.

### Immunoprecipitation and Western blotting

To examine the binding of optineurin and mutants with endogenous Rab8 and TfR, RGC5 cells were transfected with pOPTN_WT_-EGFP, pOPTN_E50K_-EGFP, pOPTN_R96L_–EGFP, pOPTN_E478G_–EGFP, pEGFP-OPTN_Q398X_, pEGFP-OPTN_2 bp-AG insertion_, and pEGFP-N1 (control) for 24 hours. Immunoprecipitation was performed using μMACS GFP Isolation Kit (MiltenyiBiotec, Auburn, CA) and MultiMACS™ M96/M96 thermo Separators (MiltenyiBiotec) following manufacturer’s protocol. In brief, cells were lysed in lysis buffer (50 mM Tris–HCl, 150 mMNaCl, 1% Triton X-100, pH 8.0) supplemented with a protease inhibitor mixture (Sigma). Clear cell lysate was incubated with 50 μl of anti-GFP microbeads to magnetically label the GFP-tagged protein for 30 min on ice. The μ column was installed on MultiMACS™ M96/M96 thermo Separators and magnetic field was applied. The μ column was equilibrated with equilibration buffer and then the lysate-microbeads mixture was loaded. After washing with 4 × 200 μl of lysis buffer and 1 × 100 μl of low salt wash buffer (20 mM Tris–HCl, pH 7.5), 20 μl of pre-heated 95°C hot elution buffer (1× SDS loading buffer) was applied to the column and incubated at room temperature for 5 min. Finally 80 μl of pre-heated 95°C elution buffer was applied to the column and elutes were collected. The elutes were subjected to SDS-PAGE under reducing conditions. The proteins were transferred to nitrocellulose membrane, and the level of co-precipitated endogenous TfR and Rab8 were assessed by mouse anti-Rab8 (BD Biosciences) and anti-TfR (Zymed Laboratories, San Francisco, CA) monoclonal antibodies. The pulled down optineurin-GFP fusion protein or GFP control was evaluated by rabbit anti-GFP polyclonal antibody (Santa Cruz Biotechnology, Santa Cruz, CA). The blot was further incubated for 1 hour with horseradish peroxidase-conjugated secondary antibody (1:10,000; Jackson ImmunoResearch Laboratories, West Grove, PA). Immunoreactive protein bands were detected by chemiluminescence using SuperSignal substrate (Pierce). Densitometry was performed. The band intensity of the co-precipitated Rab8 or TfR was normalized to that of optineurin-GFP (indicated as a ratio).

Total cell lysates from RGC5 cells transfected with pEGFP-N1, pOPTN_WT_-EGFP or pOPTN_E50K_-EGFP were also immunoprecipitated with rabbit anti-optineurin (C-Term) polyclonal antibody (Cayman Chemical, Ann Arbor, MI). Lysate of non-transfected RGC5 cells was immunoprecipitated with rabbit normal IgG as a negative control. The pulled down protein was immunoblotted with anti-TfR, anti-Rab8, or anti-optineurin. The band intensities of Rab8 and TfR were normalized to those of the endogenous optineurin or optineurin-GFP. The membranes were additionally stripped and re-probed with anti β-catenin (Santa Cruz Biotechnology) to verify the specificity of immunoblotting.

### Statistical analysis

One-way ANOVA was performed as a statistical measure for significance of the data. Statistical significance was noted if P < 0.05.

## Results

### Foci formation

**Figure 2:**
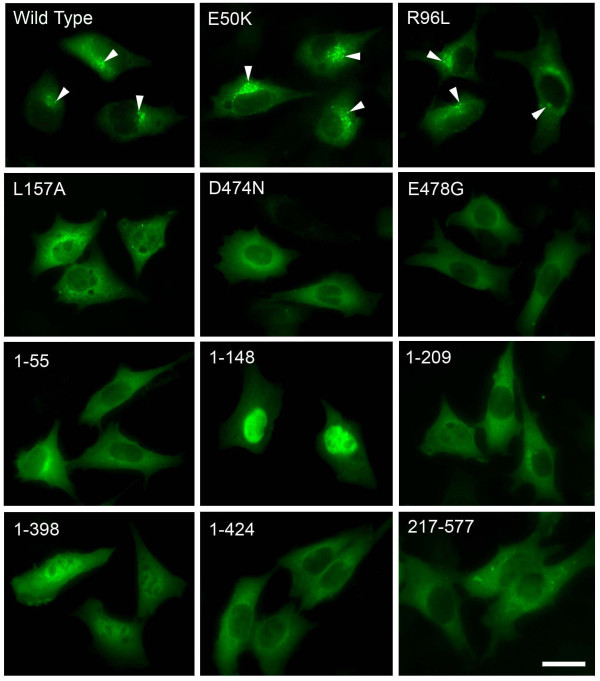
**Foci formation in RGC5 cells.** Cells transfected with pEGFP-N1 (mock control), GFP-tagged optineurin wild type, mutants as well as deletion fragment constructs were observed by fluorescence microscopy 24 hours post transfection. Bright granular structures (foci, arrowheads) in perinuclear regions were prominently observed in cells expressing optineurin wild type, E50K, and R96L, but not at all in those expressing D474N, E478G and deletion fragments 1–55, 1–148, 1–209, 1–398, and 1–424. In cells expressing mutant L157A and deletion fragments 217–577 and 425–577 (not shown), foci were typically not detected. Foci were noted occasionally, but they were small in size, low in number, and were not concentrated in the perinuclear area. Note nuclear location of deletion fragment 1–148. Scale bar, 20 μm.

**Figure 3:**
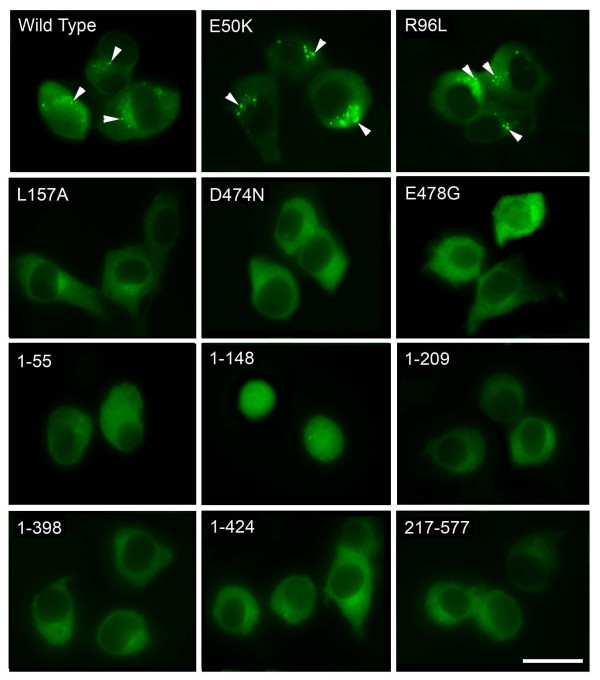
**Foci formation in Neuro2A cells.** Cells transfected with pEGFP-N1 (mock control), GFP-tagged optineurin wild type, mutants as well as deletion fragment constructs were observed by fluorescence microscopy 24 hours post transfection. Bright granular structures (foci, arrowheads) in perinuclear regions were prominently observed in cells expressing optineurin wild type, E50K, and R96L, but not in the cells transfected with other mutant or deletion constructs. Note that fragment 1–148 was expressed in the nucleus. Scale bar, 20 μm.

In RGC5 cells transfected to express wild type and E50K optineurin-GFP, bright granular structures (foci) in perinuclear regions were formed (Figure 2) as previously demonstrated (Park et al. 2006; Ying et al. 2010). R96L, located in the domain close to E50K, resulted in similarly prominent foci formation. Foci by contrast were not observed with mutations in the UBD domain, D474N and E478G. Foci were also not discerned with exon 5 deletion (1–55) and Q398X (1–398) mutations and deletion fragments 1–209, 1–424, 210–424 and 217–398 in which either the UBD or the N-terminal leucine zipper sequences were lacking or truncated (Figure 2). In cells expressing L157A, and fragments 217–577 and 425–577, foci were occasionally noted but the foci were atypical in that they were small in size, low in number, and were more spread out, not concentrated in the perinuclear area. The 2 bp-AG insertion mutant (1–148 fragment) was expressed and localized in the nucleus (Figure 2). These findings were similarly demonstrated in Neuro2A cells (Figure 3).

### Golgi fragmentation

**Figure 4:**
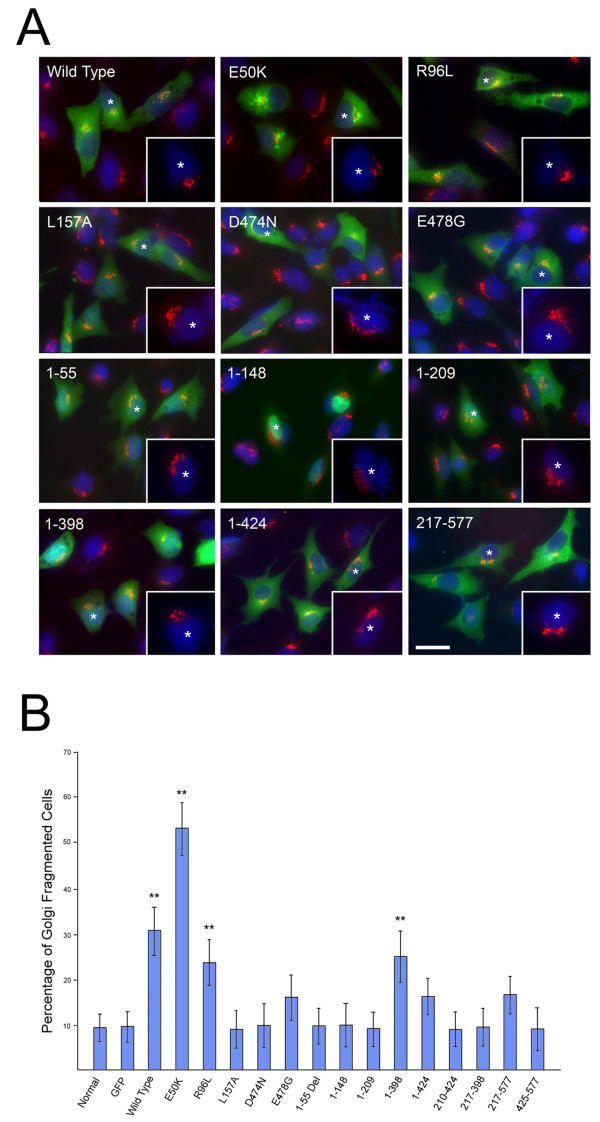
**Golgi fragmentation. A**. RGC5 cells transfected with pEGFP-N1 (mock control), GFP-tagged optineurin wild type, mutants or deletion fragment constructs were immunostained with anti-GM130 (Golgi marker, in red). Representative images of wild type, E50K, R96L, L157A, D474N, E478G, as well as deletion fragments 1–55, 1–148, 1–209, 1–398, 1–424, and 217–577 are shown. The nuclei were stained with DAPI in blue and transfected cells are in green. Severe Golgi fragmentation was observed in cells with prominent perinuclear foci formation such as wild type, E50K and R96L. The Golgi fragmentation however could still occur even without robust foci formation such as in Q398X (1–398). The percent of cells with fragmented Golgi was also increased with E478G, 1–424, and 217–577, but the values did not reach statistical significance. Higher magnification image of a transfected green cell indicated with a white star is shown in the inset. Scale bar, 10 μm. **B**. The percentage of cells with Golgi fragmentation was quantified. Compared to non-transfected normal or GFP mock controls, significantly more cells showed Golgi fragmentation in wild type, E50K, R96L and 1–398 transfected cells. **P < 0.033 compared to normal non-transfected or GFP controls. There was a moderate increase of cells with Golgi fragmentation in E478G, 1–424, and 217–577 transfected cells. All remaining mutations and deletion fragments showed no difference from controls.

To examine the integrity of the Golgi apparatus, RGC5 and Neuro2A cells were immunostained with anti-GM130, a Golgi marker. Non-transfected and GFP-expressing control cells showed robust GM130 staining and Golgi apparatus. In RGC5 cells expressing wild type, E50K, R96L, and Q398X optineurin however, the Golgi complex was disconnected, smaller, and appeared to be fragmented (Figure 4A). The percentage of cells displaying Golgi fragmentation (Figure 4B, 32.4 ± 6.1%, 55.7 ± 6.6%, 23.5 ± 5.5%. and 25.8 ± 6.1%, respectively for wild type, E50K, R96L, and Q398X optineurin) was significantly (P < 0.033) higher than that in GFP (9.8 ± 3.7%) and non-transfected (8.4 ± 3.3%) normal controls. The percentage of Golgi-fragmented cells was moderately increased in RGC5 cells overexpressing E478G (17.5 ± 6.3%), fragment 1–424 (17.4 ± 4.7%) and fragment 217–577 (18.5 ± 4.9%), although their values did not reach statistical significance. The percentages of RGC5 cells with Golgi fragmentation in L157A, D474N, exon 5 deletion (1–55), and 2 bp-AG insertion (1–148) mutants as well as 1–209, 210–424, 217–398, and 425–577 fragments were similar to controls (Figure 4B). Golgi staining of Neuro2A cells yielded comparable results (data not shown).

### Transferrin uptake

**Figure 5:**
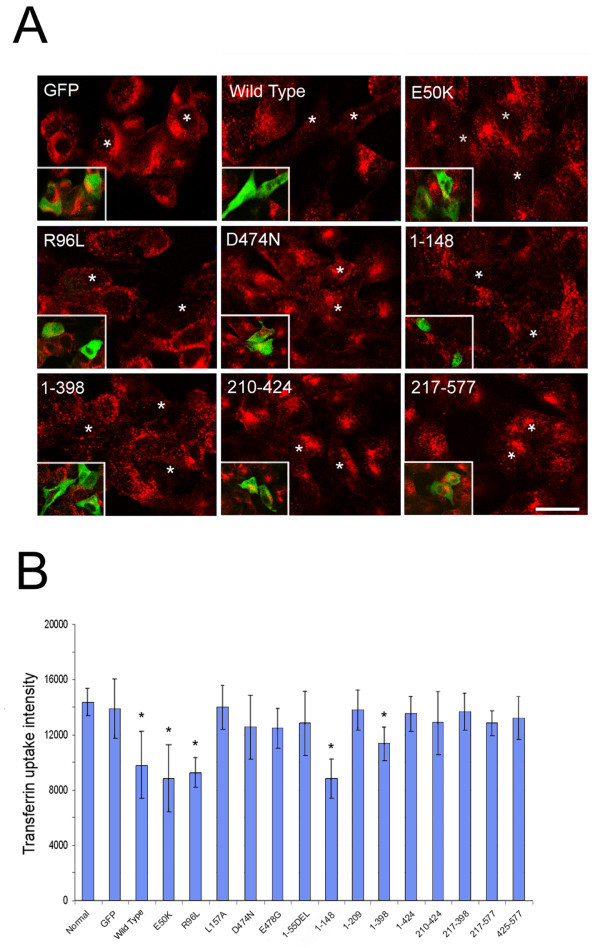
**Transferrin uptake.** RGC5 cells transfected with pEGFP-N1 (mock control) or one of the optineurin expression vectors were incubated with Texas red-transferrin (TR-Tf). Representative micrographs for GFP, and optineurin wild type (OPTN_WT_)-, OPTN_E50K_-, OPTN_R96L_-, OPTN_D474N_-, OPTN_1-148_-, OPTN_1-398_-, OPTN_210-424_-, and OPTN_217-577_-GFP expressing cells are shown **(A)**. The internalized TR-Tf is in red and the transfected cells are indicated as white stars (green channel is shown in the inset). Scale bar, 20 μm. The internalization of TR-Tf in transfected cells and non-transfected cells was quantified **(B)**. Compared to GFP mock control and non-transfected normal control, cells expressing wild type, E50K, R96L, 2 bp-AG insertion (1–148), and Q398X (1–398) optineurin had a reduced TR-Tf uptake (*, P < 0.031). The uptake impairment phenotype was not observed with E478G mutation or any other deletion fragments. Scale bar, 20 μm.

To assess the effect of forced expression of optineurin mutations and deletion fragments on transferrin uptake, RGC5 cells after transfection were incubated with TR-Tf for 15 min. The accumulation of TR-Tf in cells expressing the various constructs was shown in Figure 5A. Cells transfected with GFP had a similar level of TR-Tf red fluorescence as non-transfected cells. By comparison, cells transfected with wild type, E50K, R96L, 2 bp-AG insertion (1–148) and Q398X (1–398) optineurin had a lower TR-Tf intensity. Cells transfected with the remaining mutants and fragments, in contrast, all exhibited a similar level of fluorescence as mock control and non-transfected cells (Figure 5A).

The level of the accumulated TR-Tf in transfected cells was quantified. As shown in Figure 5B, the transferrin uptake in cells transfected with wild type and E50K optineurin was significantly decreased (P < 0.0022) compared with GFP control, corroborating previous results (Park et al. 2010; Ying et al. 2010). The impairment of transferrin uptake in cells expressing E50K (37%, average percent difference in comparison to GFP cells) was more pronounced than those expressing wild type optineurin (30%). Cells expressing R96L, 2 bp-AG insertion, and Q398X optineurin also had a significant decrease in transferrin uptake (~18–36%, P < 0.031 compared with GFP control) (Figure 5B). D474N and E478G showed only approximately 10%, non-significant reduction in transferrin uptake.

The transferrin uptake in RGC5 cells expressing L157A optineurin was unaltered (P > 0.05), in agreement with studies by Park et al. (2010). Cells transfected with the remaining optineurin fragments that lacked either the N- or C-terminal sequences or both did not exhibit any defect in the transferrin uptake (Figure 5B).

### Apoptosis

**Figure 6:**
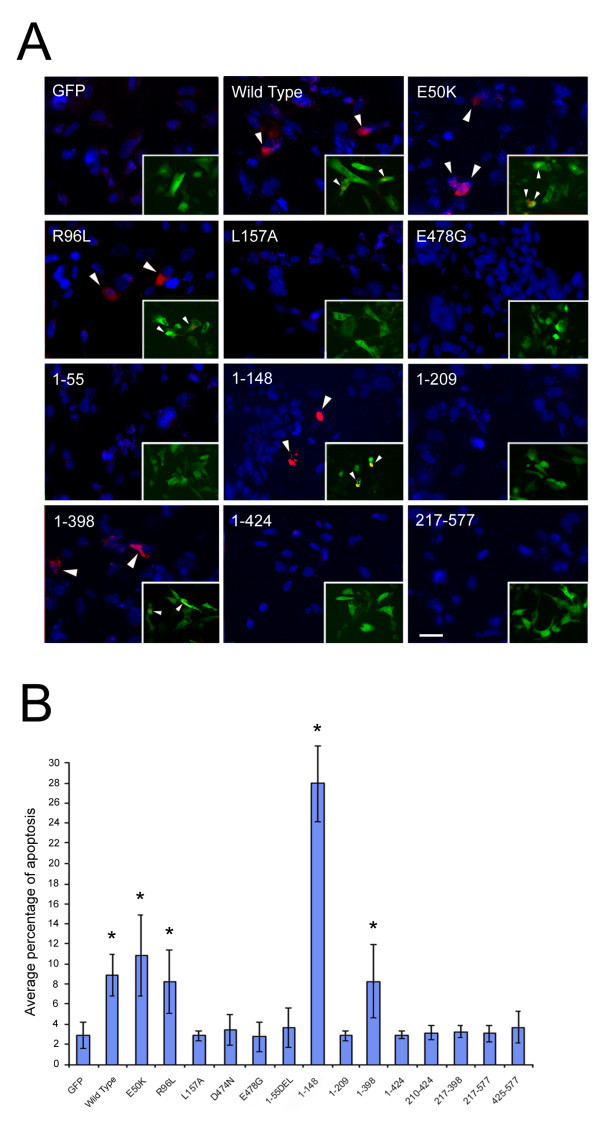
**Apoptosis.** RGC5 cells transfected with pEGFP-N1 (mock control) or one of optineurin expression vectors were incubated with caspase 3/7 substrate and Hoechst. **A**. Representative micrographs for GFP, and GFP-tagged optineurin wild type, E50K, R96L, L157A, E478G, 1–55, 1–148, 1–209, 1–398, 1–424, and 217–577 expressing cells are shown. The nuclei are stained in blue, the caspase3/7 positive cells (indicated by arrowheads) are in red and the transfected cells are in green (inset). Apoptosis was observed in cells expressing wild type, E50K, R96L, 2 bp-AG insertion (1–148) and Q398X (1–398) optineurin but not in those expressing L157A or remaining mutants and deletion fragments. Scale bar, 20 μm. **B**. Quantification of percentage of cells with apoptosis also showed similar trend as in **A**. *P < 0.0031.

Apoptosis in RGC5 cells transfected with various optineurin mutants and deletion fragments was probed with an active caspase 3/7 kit. Figure 6A shows representative images for each of the optineurin mutants and deletion fragments. Apoptotic cells were seen as active caspase 3/7-containing red fluorescent cells.

The percentage of apoptotic cells in each specimen was quantified (Figure 6B). The value was low in non-transfected and GFP mock controls (2.9% ± 1.3). Cells expressing wild type, E50K, R96L, Q398X (1–398) and 2 bp-AG insertion (1–148) optineurin all displayed significantly (P < 0.0031) increased levels of apoptotic activity (8.8 ± 2.1%, 10.8 ± 4.1%, 8.3 ± 3.1%, 8.2 ± 3.7%, 28.6 ± 11.9%, respectively) compared to GFP controls. Among them, the 2 bp-AG insertion mutant induced the highest level of apoptosis. L157A optineurin expression in cells did not show evidence of enhanced apoptosis (2.8% ± 0.6 compared with GFP control). The level of apoptosis in cells transfected with D474N and E478G mutants and the optineurin fragments remained in the normal range, similar to that of mock controls (Figure 6B).

### Levels of co-precipitated endogenous Rab8 and TfR

**Figure 7:**
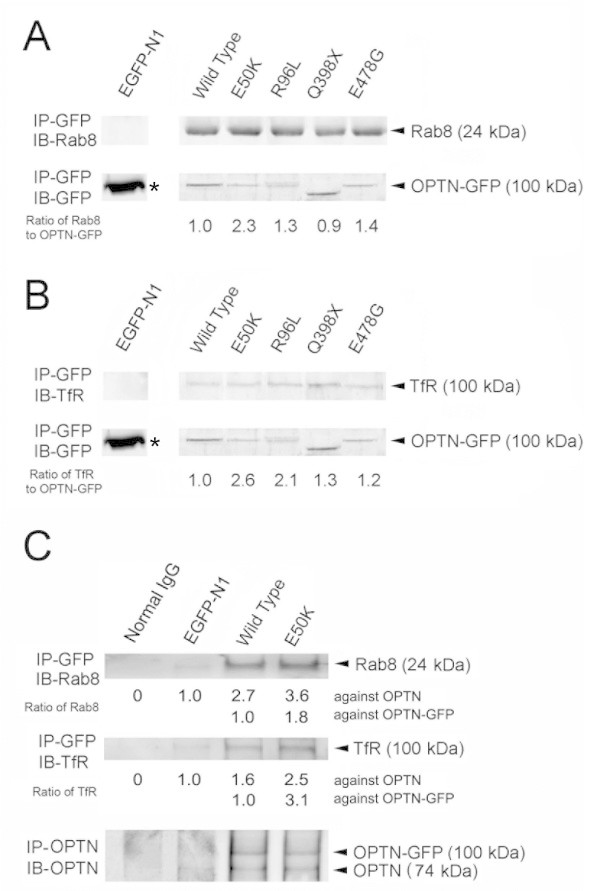
**The level of Rab8 (A) and TfR (B) co-precipitated with optineurin-GFP (OPTN-GFP).** RGC5 cells were transfected with pEGFP-N1, and GFP-tagged wild type, E50K, R96L, Q398X, and E478G optineurin constructs. OPTN-GFP was immunoprecipitated with anti-GFP microbeads. Pulled down protein was immunoblotted with anti-GFP to verify the immunoprecipitation (IP) procedure. Co-precipitated proteins were immunoblotted (IB) with anti-Rab8 **(A)**, anti-TfR **(B)**, or anti-GFP antibody. Densitometry was performed to quantify the intensity of Rab8, TfR and OPTN-GFP bands. The values were normalized against OPTN-GFP and the ratios between the mutants and wild type optineurin are presented. Note that the levels of Rab8 and TfR co-precipitated with OPTN-GFP in the mutants were similar to, or higher than, those in the wild type. As was expected, no Rab8 or TfR protein co-precipitation with GFP (25 kDa, asterisks) was detected in lysates collected from cells transfected with EGFP-N1 empty vector **(C)**. The levels of Rab8 and TfR co-precipitated with optineurin. RGC5 cells were non-transfected, or transfected with pEGFP-N1, or GFP-tagged wild type or E50K optineurin construct. Optineurin-GFP (OPTN-GFP) and endogenous optineurin (OPTN) were immunoprecipitated with anti-optineurin polyclonal antibody. Non-transfected RGC5 cell lysate was immunoprecipitated with normal IgG as negative control. The pulled down protein was immunoblotted with anti-Rab8 (top panel), anti-TfR antibody (middle panel) or anti-optineurin antibody (bottom panel, for verification of the IP procedure). Normal IgG did not yield any band for TfR or Rab8 as was expected. The ratios of Rab8/endogenous OPTN (top panel) and TfR/endogenous OPTN (middle panel) from wild type and E50K optineurin-GFP-expressing cells were higher than those of GFP control.

Rab8 and TfR have been shown to interact with optineurin (Ying et al. 2010). The level of these molecules co-immunoprecipitated with GFP in RGC5 cell lysates was examined by Western blotting. E50K-, R96L-, Q398X- and E478G-GFP fusion proteins showed more Rab8 co-pulled down than the wild type optineurin-GFP (Figure 7A). The ratio after normalization with the total amount of optineurin-GFP pulled down was: wild type (1.0), E50K (2.3), R96L (1.3), Q398X (0.9) and E478G (1.4), respectively. The mutants likewise pulled down more endogenous TfR (Figure 7B). The normalized ratio to wild type (1.0) was: E50K (2.6), R96L (2.1), Q398X (1.3), and E478G (1.2), respectively. The pull down experiment with 2 bp-AG insertion-GFP failed, likely due to the fact that this fragment was very toxic, causing severe cell death (data not shown). Negative mock control EGFP-N1, as expected, did not pull down Rab8 and TfR (Figure 7A, B).

Cell lysates of RGC5 cells transfected with pEGFP-N1, and wild type or E50K optineurin-EGFP expression vectors were also immunoprecipitated with anti-optineurin polyclonal antibody and immunoblotted with anti-Rab8, anti-TfR or anti-optineurin. The Rab8/endogenous optineurin values from wild type and E50K optineurin-GFP-expressing cells were 2.7 and 3.6 fold higher, respectively, than that of the GFP control (Figure 7C, top panel). The TfR/endogenous optineurin values were likewise higher (1.6 and 2.5 fold, respectively) in pOPTN_WT_- and pOPTN_E50K_-EGFP-transfected cells (Figure 7C, middle panel). These results indicated that relative to EGFP-N1 control or the normal situation, the Rab8 or TfR binding was increased by 2–3 fold upon overexpression of wild type and E50K optineurin-GFP. Since the Rab8 and TfR optineurin-GFP-co-precipitated levels were similar to, or greater than, those of the wild type (Figure 7A, B), mutants such as R96L and Q398X were also deduced to confer an enhanced binding capacity for these proteins.

Total cell lysate of non-transfected RGC5 cells, when immunoprecipitated with rabbit normal IgG showed no reactivity with either Rab8, TfR or optineurin antibody (negative control). The membranes were further stripped and re-blotted with an irrelevant antibody, anti-β-catenin, as an additional control. Signal was not detected on any of the blots (data not shown).

## Discussion

The current study examined the biological consequences of a series of mutations and sequence deletions in the optineurin gene to determine correlations between biomolecular functions and specific structural elements. The results presented in Figures 2, 4, 5, and 6 are summarized in Table 2.

**Table 2:** **Summary of optineurin phenotypes resulting from forced expression of optineurin mutants and deletion fragments in RGC5 cells**

	Foci formation	Golgi fragmentation	Transferrin uptake impairment	Apoptosis
Wild type	Y	Y	Y	Y
E5OK	Y	Y	Y	Y
R96L	Y	Y	Y	Y
L157A	N*	N	N	N
D474N	N	N	N	N
E478G	N	Y/N	Y/N	N
1–55	N	N	N	N
1–148	N**	N	Y	Y
1–209	N	N	N	N
1–398	N*	Y	Y	Y
1–424	N	Y/N	N	N
210–424	N	N	N	N
217–398	N	N	N	N
217–577	N*	Y/N	N	N
425–577	N*	N	N	N

Results from Figures 2, 4, 5, and 6 are summarized. Y: positive phenotype; Y/N: mild to no phenotype; N: no phenotype.

*Foci, even when formed were small in size, low in number and not in perinuclear regions.

**Nuclear localization was observed.

E50K, a mutation located in the N-terminal coiled-coil domain of optineurin, demonstrates prominent phenotypes that include foci formation, Golgi fragmentation, impairment in transferrin uptake and apoptosis. R96L, another mutation located nearby, also yielded all, albeit less pronounced phenotypes. E50K is a mutation prevalent in patients with NTG (Rezaie et al. 2002) who often suffer glaucomatous defects more severe than those without E50K mutation (Aung et al. 2005). The current data, in agreement with those published previously by our laboratory and by others (Park et al. 2006,2010; Nagabhushana et al. 2010; Kryndushkin et al. 2012), indicate that E50K is a gain-of-function mutation. A stronger-than-the-wild type interaction of the E50K mutant with Rab8 and TfR was observed (Figure 7A, B, C) (Nagabhushana et al. 2010; Park et al. 2010). We speculate that this mutation may generate a conformational change to promote protein interactions. The PROSITE analysis (Bairoch et al. 1997) however suggests that introduction of E50K mutation results in no particular change either in the structure or the conformation of optineurin (data not shown). A more definitive conclusion has to await high-resolution structural determination by X-ray crystallography. R96L, a missense mutation found in patients with ALS also appears to be a gain-of-function mutation with an enhanced binding with Rab8 and TfR (Figure 7A, B). The binding was nevertheless somewhat weaker than that seen with E50K. The weaker binding may be the basis why the R96L phenotypes were milder than those of E50K.

L157A mutation has not been identified clinically in any patients to date and is more than likely not disease causing or related. As stated above, the mutation may lead to obliteration of the leucine zipper in the optineurin sequence. L157A mutant has been shown to interact with Rab8 and TfR in a much reduced capacity (Park et al. 2010), suggesting that the optineurin association with Rab8 and TfR requires, at least in part, an intact leucine zipper motif.

No perinuclear foci were observed with L157A or with two mutations in the UBD domain, D474N and E478G. It seems that the foci formation was abrogated by mutations in either the leucine zipper or the UBD domain. Foci were also not noted when cells were transfected to express optineurin mutations/fragments with sequence deletion(s) in either or both of the domains such as 1–209, Q398X (1–398), 1–424, and 217–577. The intact sequences of both leucine zipper and UBD domains are thus concluded to be required for the perinuclear foci formation phenotype. This notion is somewhat different from that described in a recent report in which aggregates (foci) were seen in yeast with fragments 1–251, 1–398, 182–577, and 98–398 (Kryndushkin et al. 2012), as long as one of the coiled-coil domains was present. The disparity in results could be related to difference in the systems used (*in vitro* mouse cell culture versus *in vivo* yeast model), and also the definition or pattern of foci or aggregates formed.

While all optineurin phenotypes are observed when foci are formed with mutants (such as E50K and R96L), the formation does not necessarily predict the other phenotypes. One example is the fragment Q398X (1–398), which, with no foci manifestation, resulted in Golgi fragmentation, defective transferrin uptake and apoptosis. The foci are likely formed by self binding of optineurin molecules as well as their interactions with proteins including Rab8, myosin VI and TfR, requiring at least in part, intact leucine zipper and UBD motifs. Overabundance or accumulation of the protein/fragment and perturbation of the protein interactions conceivably would drive foci formation. The role or significance of the foci observed is at present uncertain. Optineurin foci are reminiscent of the inclusion bodies, Lewy bodies or aggresomes detected in neurodegenerative diseases (Grune et al. 2004). The inclusion bodies and aggresomes once were considered to be the culprit for neurodegenerative diseases. More recent evidence however suggests that they may play protective role by sequestering toxic, misfolded protein species and providing the cells with an opportunity of delayed protein degradation (Garcia-Arencibia et al. 2010; Zerovnik 2010). They may also inactivate the proteasome and mediate cytotoxicity (Glick et al. 2010). The optineurin foci may likewise have a protective role. The foci may nevertheless also signify that the cells are burdened to the extent of beyond protection. Our laboratory has shown that the proteasome activity is compromised and autophagy is induced when cells overexpress wild type and E50K optineurin (Shen et al. 2011). A very recent study on another optineurin variant, M98K, has further linked the autophagic process to apoptosis in RGC5 cells (Sirohi et al. 2013).

It was speculated (Ying and Yue 2012) that alterations in the interaction between optineurin and its binding partners (such as Rab8, myosin VI, and Htt) may disturb the balance between actin- and microtubule-based motor systems and contribute to fragmentation of the Golgi complex. Consistent with this hypothesis, Golgi fragmentation was noted with wild type optineurin and mutants E50K, R96L and Q398X (or fragment 1–398), all of which demonstrated an enhanced Rab8 binding capacity (Figure 7A, C). E478G mutant that resulted in Golgi fragmentation at a mild level (Figure 4) also showed stronger binding with Rab8 (Figure 7A). With deletion in the N-terminal sequences (fragment 217–577) and deletions of the C-terminal Htt or myosin VI-binding sequences (fragment 1–424), mild Golgi fragmentation was likewise observed (Figure 4). However, when both the N-terminal Rab8-binding and the C-terminal protein binding domains were missing (fragments 1–55, 210–424, and 217–398), no Golgi fragmentation resulted. Exceptions however were seen that include D474N mutant and fragments 1–209 and 425–577. It is possible that in those situations, the binding disturbance was not sufficient to induce any Golgi defect.

A heightened optineurin binding with TfR has been implicated as a factor leading to impairment of the transferrin uptake (Park et al. 2010). Apoptosis may ensue either from the trafficking impairment and/or the Golgi fragmentation. In accordance with these hypotheses, wild type optineurin and E50K, R96L, and Q398X (1–398) mutants that confer the transferrin uptake phenotype all exhibit a strong binding capacity with TfR (Figure 7B, C). This same set of optineurins also induced apoptosis in cells, suggesting that the protein trafficking phenotype may be correlated with apoptosis, more so than the Golgi fragmentation. Of note is that fragment 1–424, contrasting Q398X, did not impact transferrin uptake or apoptosis level. This fragment is only 26 amino acid residues longer than Q398X. Perhaps addition of sequences between residues 399 and 424 enables the protein to adopt into a different, non-consequential conformation. E478G had an elevated ratio of TfR co-precipitated with optineurin-GFP (with a ratio of 1.2 relative to the wild type, Figure 7B) but provoked only about 10%, non-significant reduction in the transferrin uptake (Figure 5B).

The nonsense 691_692insAG or the optineurin fragment 1–148 (also called c.382_383insAG or 2 bp-AG insertion) mutation that presumably induces a premature stop codon in exon 6, stands out from others as it has a nuclear localization. Clinically, it was identified both in patients with glaucoma (Rezaie et al. 2002) and a patient with young ALS onset (46 years) and rapid disease progression (21 months) (Millecamps et al. 2011). Optineurin does not contain nuclear localization sequences. It has however been reported that optineurin is translocated to the nucleus in response to oxidative stress (De Marco et al. 2006). At mitotic entry, Plk1 phosphorylates optineurin and dissociates it thereby from the Golgi-localized Rab8, and induces its translocation to the nucleus (Kachaner et al. 2012a). While the mechanism is unclear, the finding that the 2 bp-AG insertion variant localizes in the nucleus indicates that the localization determinants reside in the first 148 N-terminal sequences. This mutation is highly toxic, drastically raising the level of apoptosis in cells (Figure 6). The cause of cell death, seemingly unrelated to either the Golgi or the transferrin uptake defect, remains to be determined.

E478G, a missense mutation in the UBD region, is associated with ALS. Patients with heterozygous E478G mutation had later onset with slower progression (Ito et al. 2011). It was suggested that the E478G mutant might have a dominant-negative effect. Consistent with our data, optineurin inclusion bodies were not prominent in the patients. A mild case of Golgi fragmentation was observed (Ito et al. 2011; Figure 4). The E478G mutation in the UBD domain may conceivably interfere with NF-κB and anti-viral signaling, leading to pathology under stress conditions such as inflammation and pathogen invasion. It is on the other hand, unclear why mutation D474N, also at the UBD domain very close to the E478G mutation, does not manifest any phenotypes and is not disease-associated so far. Again, a detailed, high-resolution 3-dimensional optineurin structure will be required for a clearer perspective.

RGC5, an immortalized cell line established by transforming postnatal day 1 rat retinal cells with E1A adenovirus (Aoun et al. 2003; Agarwal 2013) has been used widely and extensively as a model of RGC for various investigations (Harvey and Chintala 2007; Yang et al. 2009). A re-characterization by Van Bergen et al. (2009) utilizing both mitochondrial and nuclear DNA analysis however led to the conclusion that this cell line was of mouse origin rather than rat. More recently, an investigation by the original RGC5 cell line creator Krishnamoorthy et al. (2013) presented evidence indicating that the RGC5 cell line shared approximately 95% (60/63 of total) of genetic markers with a mouse derived photoreceptor cell line (661W). In the present study, in addition to RGC5 cells, we also evaluated the foci formation and Golgi fragmentation phenotypes in a mouse brain neuroblastoma Neuro2A cell line. Results similar to those from RGC5 cells were observed (Figure 3). Previously, our group has likewise demonstrated optineurin phenotypes in other wild type and E50K optineurin-expressing cells including human retinal pigment epithelial, human trabecular meshwork and rat neuronal PC12 cells (Park et al. 2006,2010; Koga et al. 2010; Shen et al. 2011). We thus surmise that the phenotypes may not be cell type dependent, but likely represent cellular changes induced by optineurin expression or mutations.

In summary, the current study correlates the biologic consequences with structural elements in the optineurin gene. Lending support to previous investigations, our results depict that optineurin exerts different functions and impacts various biologic processes through interactions with other proteins. The study provides clues as to what regions of the gene may play a critical role in the development of pathologic consequences. It also underscores the need of X-ray crystallography work for the 3-dimensional structure to analyze conformational changes in relation to biomolecular functional consequences induced by mutations or truncations.
